# Procedural justice and egalitarian principles for rationing decisions in the COVID-19 crisis

**DOI:** 10.1186/s13054-020-03283-w

**Published:** 2020-09-29

**Authors:** Alexander Supady, Christoph Bode, Daniel Duerschmied

**Affiliations:** 1 Department of Medicine III (Interdisciplinary Medical Intensive Care), Medical Center – University of Freiburg, Faculty of Medicine, University of Freiburg, Freiburg, Germany; 2 Department of Cardiology and Angiology I, Heart Center, University of Freiburg, Hugstetter Strasse 55, 79106 Freiburg, Germany; 3 Heidelberg Institute of Global Health, University of Heidelberg, Heidelberg, Germany

In their insightful editorial on the justification for extracorporeal membrane oxygenation (ECMO) treatment in the coronavirus disease 2019 (COVID-19) pandemic, Abrams et al. argue for a utilitarian principle “reserving ECMO only for those patients who are most likely to derive benefit”—and consequently withholding ECMO from others with less desirable prospects [1]. We doubt that the utilitarian approach is appropriate in this context; therefore, we would like to suggest an alternative approach.

Norman Daniels developed “a theory of justice and health” that may help to make fair resource allocation decisions for ECMO during the COVID-19 pandemic [2]. In contrast to allocating resources solely guided by expected outcomes and maximization of net utility, Daniels proposes a focus on a fair process for making resource allocation decisions. Characteristic elements of this process called “accountability for reasonableness” are transparency and participation. In short, decisions and their rationales must be publicly accessible (*publicity condition*), they must be based on objective evidence or reasons and principles generally accepted as relevant (*relevance condition*), mechanisms for appeal and revision of decisions need to be in place (*revision and appeals condition*), and mechanisms to control and guarantee compliance with these principles must be established (*regulative condition*) [2].

Adoption of these principles for triage for or against ECMO therapy during the COVID-19 pandemic will help to unburden physicians from rationing decisions when treating their patients and to raise acceptance for necessary limitations. The practical application of these principles can well be done by triage committees as being installed in New York [3]. As described previously, members of these committees must be free of competing interests; this includes that they cannot be involved in the treatment of the patient the committee decides about. However, it seems equally necessary to set up these committees in such a way that their decisions are public and transparent and allow for public participation—the role of juries or lay assessors in court may serve as an example to find a structure fulfilling these demands.

It is not possible within the limited scope of this letter to describe a comprehensive account for fair setting of health care limits in a crisis like the coronavirus pandemic nor to describe in detail a fair process for limit setting and triage for ECMO therapy in COVID-19, but we encourage further thoughts about Daniels’ “accountability for reasonableness” as a basis for deliberations on a fair distribution of limited health care resources.

## Data Availability

Not applicable.
