# Supplementary material for: Procedural justice and egalitarian principles for rationing decisions in the COVID-19 crisis
Source: Crit Care. 2020 Sep 29;24:590. doi: 10.1186/s13054-020-03283-w (PMC7524376; doi:10.1186/s13054-020-03283-w)
Supplement: Related texts

# Reply: Authors’ response

**Authors:** Darryl Abrams, Roberto Lorusso, Jean-Louis Vincent,, Daniel Brodie

We thank the authors for their thoughtful reply to our article “ECMO during the COVID-19 pandemic: when is it unjustified?” [1], in which they suggest the use of triage committees to ensure fair allocation of ECMO during the COVID-19 pandemic, an issue we raised in our article. We also appreciate the greater focus given to this important issue by the authors. Their intention of designing a system emphasizing transparency and objectivity of decision making is laudable. However, its use in allocating ECMO for COVID-19-associated severe respiratory failure would be complicated and in many ways may prove to be impractical. This is particularly clear during the resource constrained stage of a pandemic, where crisis standards of care are in place and precisely when such a system would be needed. Importantly, the triage committees the authors cite as having been deployed in New York [3] were never actually put in place because of the controversy they generated and the difficulty meeting the conditions set out by the authors as a framework for resource allocation, speaking volumes about the complexity of the undertaking. In jurisdictions where they were attempted, they were met with controversy and lawsuits [4].

If, after years of preparation for just such a scenario, triage committees in many instances could not be successfully implemented for allocation of ventilators, which are a widely accepted form of life support, then doing the same for ECMO, where agreement regarding indications and contraindications is less well established particularly in COVID-19 (the “relevance condition” cited by the authors), would be even more unlikely. We agree with the authors that, ideally, we should strive for a method of equitably distributing limited healthcare resources during a crisis, such as that proposed by Daniels, although doing so will clearly require a triage system that is both workable and acceptable to society at large. It will also require a thorough understanding of the benefits and limitations of the resource being considered, a condition that would be difficult to meet in the case of ECMO at the present time. In the end, a faulty triage plan based on insufficient data may be worse than no triage plan at all [5].
